# Electrochemically Synthesized Nanoporous Molybdenum Carbide as a Durable Electrocatalyst for Hydrogen Evolution Reaction

**DOI:** 10.1002/advs.201700601

**Published:** 2017-12-19

**Authors:** Jin Soo Kang, Jin Kim, Myeong Jae Lee, Yoon Jun Son, Dong Young Chung, Subin Park, Juwon Jeong, Ji Mun Yoo, Heejong Shin, Heeman Choe, Hyun S. Park, Yung‐Eun Sung

**Affiliations:** ^1^ Center for Nanoparticle Research Institute for Basic Science Seoul 08826 Republic of Korea; ^2^ School of Chemical and Biological Engineering Seoul National University Seoul 08826 Republic of Korea; ^3^ School of Advanced Materials Engineering Kookmin University Seoul 02707 Republic of Korea; ^4^ Fuel Cell Research Center Korea Institute of Science and Technology (KIST) Seoul 02792 Republic of Korea

**Keywords:** anodization, carbon shells, electrocatalysts, hydrogen evolution, molybdenum carbide

## Abstract

Demands for sustainable production of hydrogen are rapidly increasing because of environmental considerations for fossil fuel consumption and development of fuel cell technologies. Thus, the development of high‐performance and economical catalysts has been extensively investigated. In this study, a nanoporous Mo carbide electrode is prepared using a top‐down electrochemical process and it is applied as an electrocatalyst for the hydrogen evolution reaction (HER). Anodic oxidation of Mo foil followed by heat treatment in a carbon monoxide (CO) atmosphere forms a nanostructured Mo carbide with excellent interconnections, and these structural characteristics lead to high activity and durability when applied to the HER. Additionally, characteristic behavior of Mo is observed; metallic Mo nanosheets form during electrochemical anodization by exfoliation along the (110) planes. These nanosheets are viable for chemical modification, indicating their feasibility in various applications. Moreover, the role of carbon shells is investigated on the surface of the electrocatalysts, whereby it is suggested that carbon shells serve as a mechanical barrier against the oxidative degradation of catalysts that accompanies unavoidable volume expansion.

## 1 Introduction

Recent advances in fuel cell technology have increased expectations for practical utilization of hydrogen as an energy source.^1^, ^2^, ^3^, ^4^, ^5^, ^6^, ^7^ Intensive investigations on sustainable production of H_2_ by water electrolysis have been carried out,^8^, ^9^, ^10^ especially to develop electrocatalysts to facilitate the hydrogen evolution reaction (HER).^11^, ^12^, ^13^ The catalytic activity of platinum is incomparably high for the HER,^14^, ^15^ but it is unfavorable for commercial applications because it is rare and expensive.^12^ Earth‐abundant transition metal compound such as carbides,^16^, ^17^ nitrides,^18^, ^19^ sulfides,^20^, ^21^, ^22^, ^23^ selenides,^24^, ^25^ and phosphides^26^, ^27^, ^28^, ^29^ was recently introduced as more practical and realistic electrocatalysts for the HER, and remarkably high performances were achieved using numerous compound materials; however, further enhancement in stability is essential for their practical use in acidic media.^30^, ^31^, ^32^

Among the economical electrocatalysts, carbides have exhibited exceptional activity because of their favorable electronic structure that originates from the modification of the d‐band structure induced by metal–carbon bond formation.^11^, ^17^, ^33^ Mo_2_C is a typical example that shows high performance in the HER, and substantial advances have been accomplished by structural and combinatorial modifications of Mo_2_C electrocatalysts.^16^, ^34^, ^35^, ^36^ Carbide synthesis is often accompanied by severe sintering of the particles, which increases the size of the carbide catalysts and reduces the performance. Until recently, nanostructured Mo_2_C catalysts have been synthesized with the assistance of carbonaceous materials to prohibit agglomeration of nanoparticles.^36^, ^37^, ^38^

**Figure 1:**
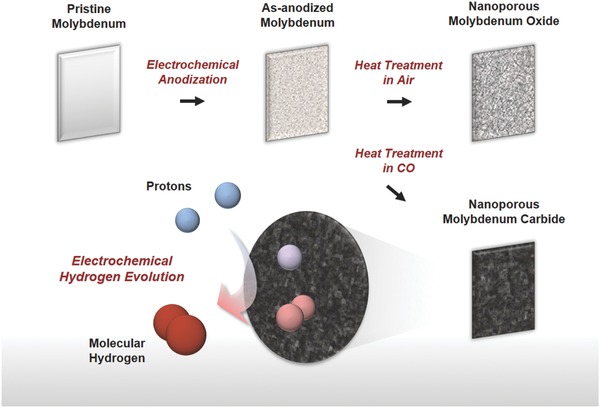
Schematic images summarizing the procedures of electrochemical synthesis of nanoporous Mo carbide and its application in electrochemical hydrogen evolution.

In this study, a nanoporous Mo carbide electrode was prepared via electrochemical anodization followed by heat treatment in a CO atmosphere; the nanoporous Mo carbide electrode was directly applied as an electrocatalyst for the HER (see the schematic summary in **Figure** **1**). This method enabled synthesis of nanostructured carbide catalysts without using a template or carbonaceous agent but formed an Mo_2_C electrode with a thoroughly interconnected nanostructure and extremely thin (≈1 nm) carbon shells on its surface. This nanoporous Mo carbide electrode exhibited high activity and excellent durability for the HER, which led to a negligible performance drop even after 3000 cycles of the accelerated durability test (ADT). Furthermore, we observed the characteristic behavior of Mo in anodic oxidation and the role of carbon shells in long‐term HER operation. Metallic Mo sheets were formed during the anodization by exfoliation of the (110) plane; this is different from the self‐ordering phenomenon seen in other anodic oxides. These Mo nanosheets were easily transformed into nanostructured compounds by postmodification, implying the feasibility of anodic Mo compounds in various applications. In addition, an investigation into the role of carbon shells was performed via ex situ electron microscopy and X‐ray analyses. Based on the experimental observations, we herein suggest that carbon shells on the surface of electrocatalysts serve as a mechanical barrier against the oxidative degradation of electrocatalysts that accompanies volume expansion.

## 2 Results and Discussion

### 2.1 Electrochemical Synthesis of Nanoporous Molybdenum Carbide

**Figure 2:**
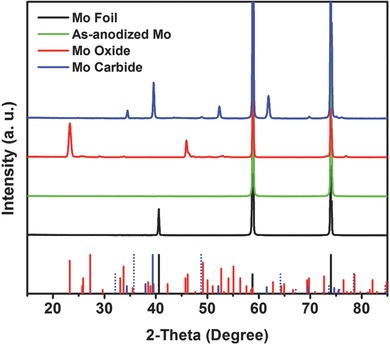
XRD patterns of Mo foil, as‐anodized Mo, Mo oxide, and Mo carbide. The signals were assigned in accordance with the reference 2‐theta positions of Mo (black bars, JCPDS 01‐1208), MoO_3_ (red bars, JCPDS 05‐0506), MoC (blue dotted bars, JCPDS 45‐1015), and Mo_2_C (blue bars, JCPDS 35‐0787).

Electrochemical anodization of Mo foils was carried out at 40 V for 2 h at 25 °C using an ethylene glycol electrolyte containing 0.25 wt% NH_4_F, 2 vol% H_2_O, and 0.1 M NaOH. NaOH was added to create mild anodization conditions for Mo by reducing the local pH drop that is responsible for chemical dissolution of anodic oxides.^39^ For the preparation of Mo carbides, as‐anodized Mo foils were heat treated at 800 °C for 4 h in a carbon monoxide (CO)‐filled tubular furnace, and crystalline Mo oxide was also prepared for comparison by thermal annealing at 450 °C for 4 h in air to gain a deeper understanding of the anodization of Mo and further modifications. The formations of crystalline oxides and carbides were characterized by obtaining X‐ray diffraction (XRD) patterns (**Figure** **2**). For the as‐anodized Mo foil, the only crystalline phase signal is from the metallic Mo substrate (JCPDS 01‐1208), while MoO_3_ peaks (JCPDS 05‐0506) are clearly apparent after thermal annealing in air. Heat treatment in CO formed carbides identified as Mo_2_C (JCPDS 35‐0787) with a small amount of MoC (JCPDS 45‐1015) as a secondary phase. Meanwhile, the Mo (110) peak is not present after anodization, and it is undetectable in the crystalline Mo oxide and carbide samples after the postheat treatment.

**Figure 3:**
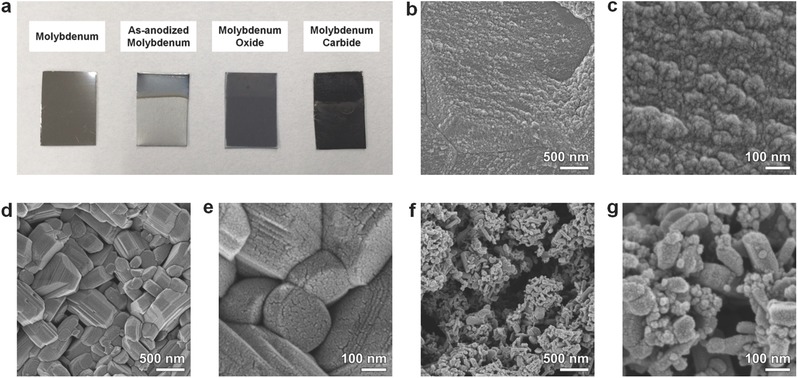
a) Digital images of Mo foil and anodized Mo before and after heat treatments in air (oxide) or a CO atmosphere (carbide). SEM images of b,c) as‐anodized Mo, d,e) Mo oxide, and f,g) Mo carbide.

**Figure** **3**a shows a digital photograph image of the pristine Mo foil, the as‐anodized Mo, and the crystalline Mo oxide and Mo carbide synthesized by heat treatment in air and a CO atmosphere, respectively. The microstructures of the electrochemically synthesized Mo compounds were characterized by scanning electron microscope (SEM), and Figure 3b,c shows SEM images of the as‐anodized Mo foil, which was then transformed into crystalline Mo oxides (Figure 3d,e) and Mo carbides (Figure 3f,g). Unlike the as‐anodized Mo, the Mo oxides, and carbides have characteristic nanostructures. From these observations, we noticed that the nanostructures form during the heat treatment rather than during electrochemical anodization, though their dimensions are significantly different in the oxide and carbide. These samples are denoted as np‐MoO_3_ and np‐Mo_2_C from the nanoporous structures and phases (characterized by XRD analysis) of the crystalline Mo oxide and carbide, respectively. The thicknesses of the np‐MoO_3_ and np‐Mo_2_C layers on the Mo substrates were measured from the cross‐sectional SEM images (see Figure S1, Supporting Information) and are ≈2 µm for both.

**Figure 4:**
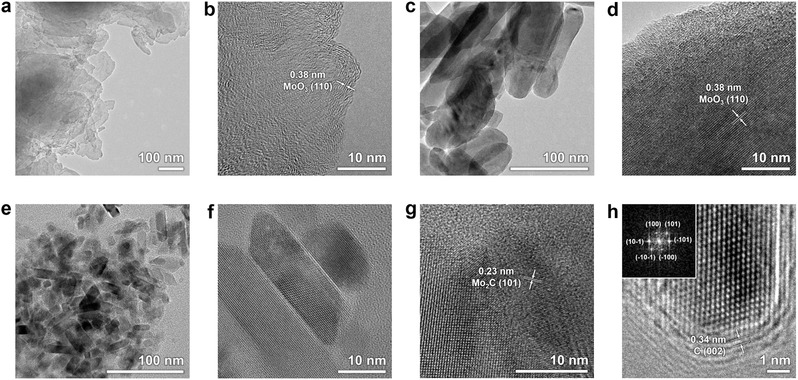
TEM images of a,b) as‐anodized Mo, c,d) Mo oxide, and e–h) Mo carbides. The inset in (h) shows the FFT patterns.

Transmission electron microscope (TEM) images were obtained to investigate the nanostructures and crystallinity of the Mo compounds. **Figure** **4**a,b shows that the surface of the as‐anodized Mo foil is composed of nanosheets. Partially crystalline phases are observed at the edges within ten monolayers, and this is characterized as MoO_3_ from the lattice spacing of 0.38 nm corresponding to the (110) plane. Based on the XRD result where the (110) peak of the Mo metal is diminished after anodization, and the fact that the as‐anodized Mo had sheet‐like morphologies, we could perceive that the exfoliations occur at the (110) plane of Mo during the electrochemical anodization (this phenomenon is discussed below in more detail). After the heat treatment in air, a crystalline oxide of several hundreds of nanometers was formed (np‐MoO_3_), as displayed in Figure 4c. The high‐magnification TEM image shows a lattice spacing of 0.38 nm (Figure 4d), which matches the (110) plane of MoO_3_. The TEM images in Figure 4e–h show the morphologies of np‐Mo_2_C, of which sizes are approximately tens of nanometers. The high‐magnification image displayed in Figure 4g and the (101) planes of the Mo_2_C crystals with d‐spacings of 0.23 nm are apparent. The crystalline Mo_2_C phase was further confirmed by fast Fourier transform (FFT) analysis (see Figure 4h).

Additionally, it is notable that thin carbon shells with a thickness of around 1 nm are present on the surface of np‐Mo_2_C. Formation of carbon layers has been frequently observed in a number of previous studies wherein carbide materials were synthesized by heat treatment in a gaseous precursor‐filled chamber.^40^, ^41^ The carbon shells seem to have originated from the chars from the pyrolysis of C‐containing gases.^42^, ^43^ It was reported that around 50% of activity drop occurs when the graphitic carbon on the catalyst's surface is composed of three to four monolayers.^40^ Meanwhile, carbon shells are known to enhance the stability of electrocatalysts by suppressing degradation.^40^, ^44^, ^45^ Therefore, an enhancement in stability and loss in catalytic activity was expected for np‐Mo_2_C due to the presence of carbon shells on the surface.

**Figure 5:**
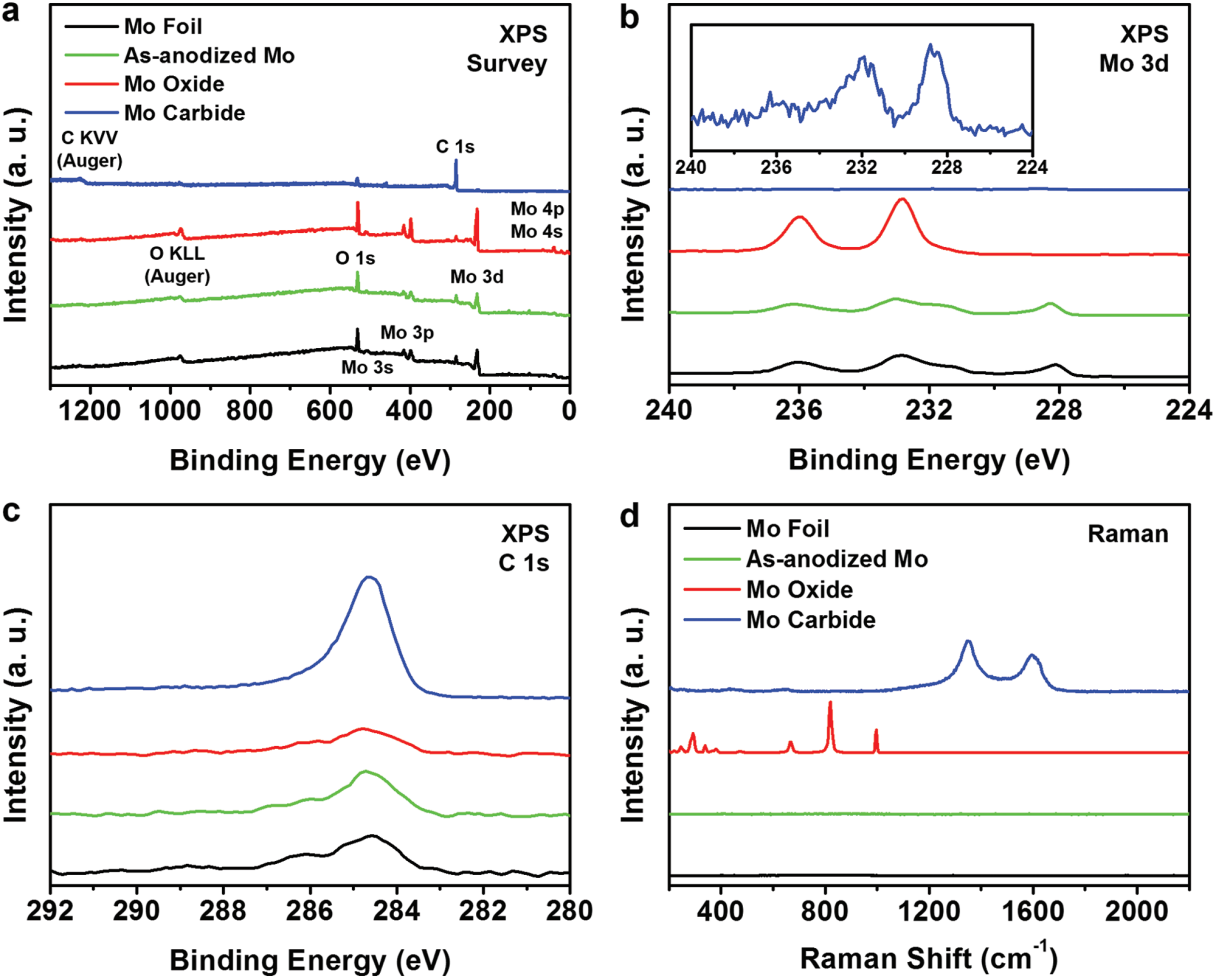
XPS a) survey, b) Mo 3d, and c) C 1s spectra, and d) Raman spectra of Mo foil, as‐anodized Mo, Mo oxide, and Mo carbide. The inset in (b) shows the enlarged XPS Mo 3d spectrum of Mo carbide.

We performed X‐ray photoelectron spectroscopy (XPS) to characterize the surface states of the synthesized Mo compounds. In the XPS survey spectra displayed in **Figure** **5**a, all of the peaks were able to be assigned as signals from Mo, O, and C. Figure 5b shows Mo 3d spectra of the Mo compounds, and several noteworthy observations were made. For the Mo foil, peaks from metallic Mo are observed at binding energy (BE) positions of 228.2 and 231.4 eV, and those from the oxidized surface are apparent at 232.7 and 235.9 eV.^46^, ^47^ Interestingly, signals from metallic Mo are detected for as‐anodized Mo, and the overall shape of its Mo 3d spectrum is similar to that of the Mo foil. This indicates that in our case, the surface of Mo foil is unlikely to be transformed into Mo oxides during the anodization process. In contrast, only the strong Mo oxide peaks are observed after thermal annealing in air. These results imply that the oxidation of Mo takes place during the heat treatment step rather than during the electrochemical anodization step. Meanwhile, signals at 228.7 and 231.9 eV are detected for np‐Mo_2_C, and these results correspond to the BE positions of Mo_2_C.^48^, ^49^ Compared to other samples, the Mo 3d peak intensities are smaller in np‐Mo_2_C, probably due to the presence of carbon shells on the surface, and this can be supported by the substantially large C 1s signal of np‐Mo_2_C (Figure 5c). From the Raman spectra displayed in Figure 5d, the chemical identity of np‐MoO_3_ is crosschecked by the signals that matched exactly with previous reports on MoO_3_, including the high‐intensity peaks at 819 cm^−1^ (terminal Mo—O stretching) and 993 cm^−1^ (bridge Mo—O—Mo stretching).^50^, ^51^ In addition, the existence of graphitic carbon layers on np‐Mo_2_C is clearly verified by the D and G signals at 1350 and 1580 cm^−1^, respectively.^52^ Another noteworthy observation from the Raman analysis was that the as‐anodized Mo did not exhibit any response. Together with the XPS spectra, where almost identical signals were obtained from the pristine and anodized Mo foils, the Raman spectra verify that the nanosheets formed during anodic oxidation are metallic Mo.

### 2.2 Characteristic Behavior of Molybdenum during Anodic Oxidation

As mentioned above, Mo exhibits an interesting behavior during the electrochemical anodic oxidation process: metallic Mo sheets are formed by exfoliations at the (110) planes. In general, nanostructured anodic oxides develop during anodization, and thermal annealing in air is merely responsible for increasing the crystallinity. In addition, chemical transformations by heat treatment in a gaseous precursor‐filled atmosphere seldom trigger significant changes in the overall nanostructures. These phenomena have been reported for various materials, such as Al,^53^ Ti,^54^, ^55^ Fe,^56^, ^57^ Sn,^58^, ^59^ and W.^60^, ^61^ In contrast, anodization‐induced exfoliation at the (110) planes of Mo results in the Mo nanosheets with partially oxidized edges. These anodic sheets are feasible for postmodification into different chemical compositions and morphologies depending on the heat treatment conditions. In our case, as‐anodized Mo is rearranged into the nanostructured oxide or carbide during the heat treatment in air or CO atmosphere, respectively.

**Figure 6:**
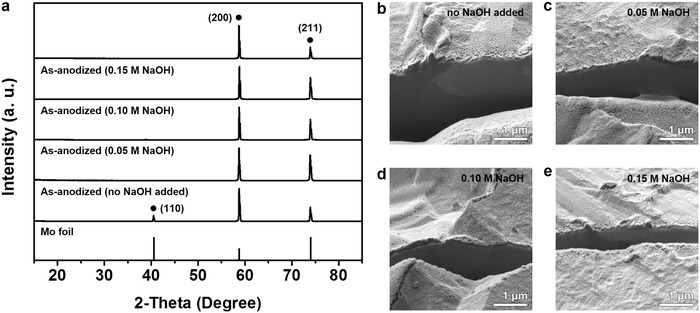
a) XRD patterns of as‐anodized Mo foils when electrolytes containing 0–0.15 m of NaOH were used. The signals were assigned with black circles in accordance with the reference 2‐theta positions of Mo (black bars, JCPDS 01‐1208). SEM images of as‐anodized Mo foil when electrolytes containing b) none, c) 0.05 m, d) 0.10 m, or e) 0.15 m of NaOH was used. The SEM images were taken after the focused ion beam milling.

We performed further investigations to determine whether this phenomenon is caused by our specific experimental condition. Since it is well known that F^−^‐containing compound (e.g., NH_4_F or NaF) and H_2_O are necessary for electrochemical anodization in viscous organic electrolytes,^39^, ^62^ we investigated the effect of the other additive—NaOH—on the exfoliation of Mo. **Figure** **6**a shows the XRD patterns of as‐anodized Mo foils prepared by using the electrolyte with different NaOH contents (none, 0.05, 0.10, and 0.15 m). Exfoliation at the (110) plane is recognizable in all cases by the diminished Mo (110) peak. Moreover, negligible difference in morphology is observable from the SEM analyses (see Figure 6b–e). It was also verified that the disappearance of the Mo (110) peak does not originate from the postheat treatment, which is confirmed by observing the presence of the Mo (110) peak from the XRD pattern of the heat‐treated (450 °C in air) Mo foil (see XRD patterns in Figure S2, Supporting Information). Recently, Schmuki and co‐workers reported the synthesis of nanotubular Mo oxide by using a viscous electrolyte with extremely high NH_4_F and H_2_O contents,^63^ and in concurrence with this, we are able to conclude that exfoliation of Mo to produce metallic nanosheets during anodic oxidation is a characteristic behavior of Mo, especially under mild anodization conditions.

### 2.3 Application of Nanoporous Molybdenum Carbide in Electrocatalytic Hydrogen Evolution Reaction

**Figure 7:**
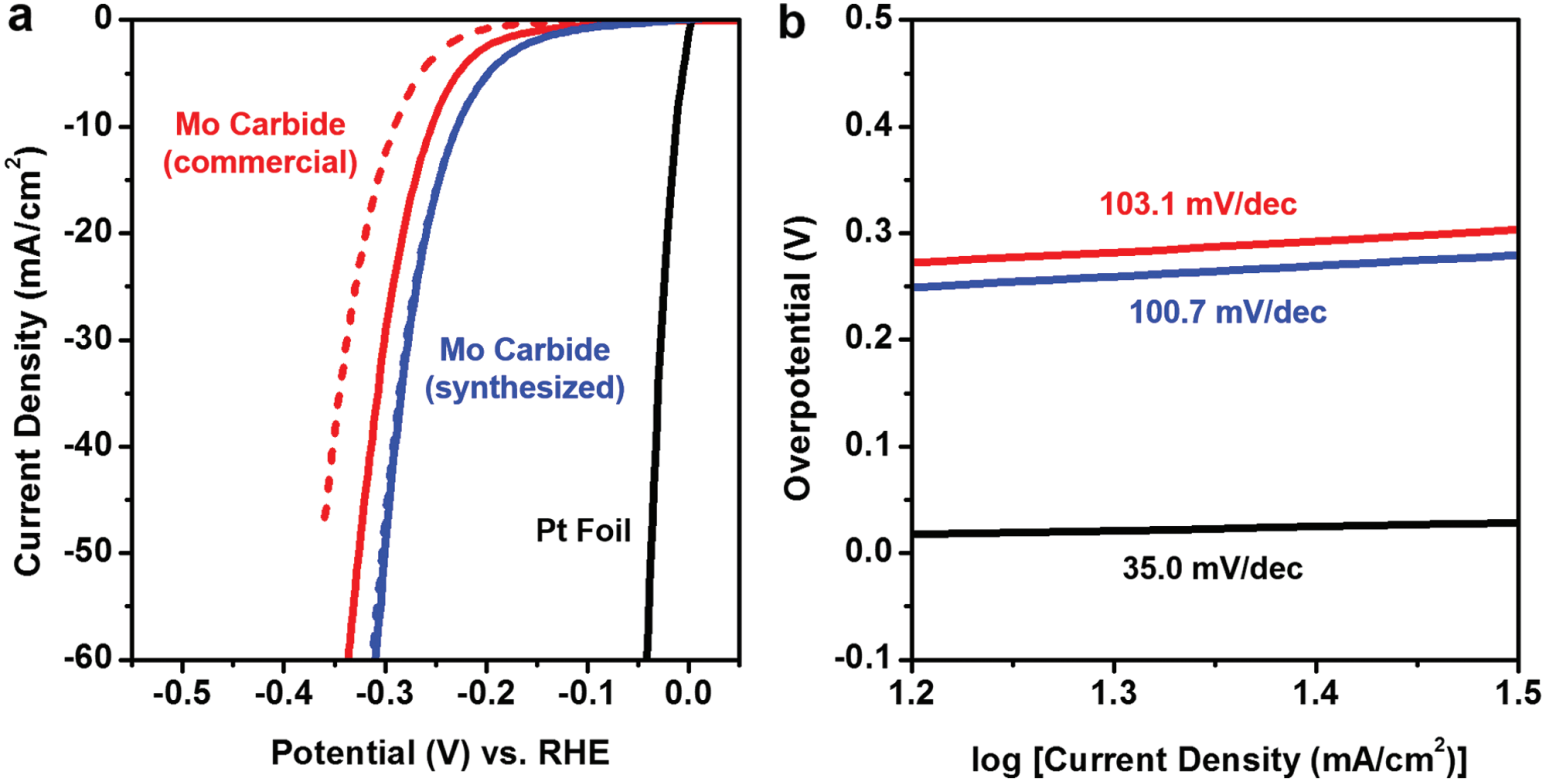
a) iR‐corrected HER polarization curves and b) Tafel plot of Pt foil, commercial Mo carbide, and electrochemically synthesized Mo carbide. The electrochemical measurements were performed in 0.5 m H_2_SO_4_ solution, at the scan rate of 20 mV s^−1^ and rotation speed of 2000 rpm. The solid and dashed curves in (a) respectively show the HER currents before and after the 3000 cycles of ADT, which was carried out at the scan rate of 100 mV s^−1^. The values in (b) indicate the Tafel slopes.

Electrochemical anodization of Mo followed by postfunctionalization forms a nanostructured Mo carbide film with high uniformity and connectivity. In addition to the benefits originating from the nanomorphologies, np‐Mo_2_C was expected to have an excellent durability in various electrocatalyses because polymeric binders and carbonaceous conducting agents are not required for the electrode preparation due to its interconnected nanostructure. Moreover, the presence of thin carbon shells on the surface is known to effectively suppress any possible degradations.^64^, ^65^, ^66^, ^67^ Since Mo carbide is known for its favorable electronic structure for catalyzing electrochemical HER,^36^ we applied np‐Mo_2_C as an electrocatalyst for hydrogen evolution in an acidic medium (0.5 m H_2_SO_4_ solution). **Figure** **7**a shows the iR‐corrected HER polarization curves of Pt foil, commercial Mo_2_C (chemical composition was confirmed by XRD, see Figure S3, Supporting Information), and np‐Mo_2_C prepared by the electrochemical method. Compared with Pt, which manifests a steep increase in the HER current when the potential is below 0 V versus reversible hydrogen electrode (RHE), both of the Mo_2_C show moderate performances. The overpotential to achieve 10 mA cm^−2^ is 12 mV for Pt, but 229 and 254 mV are needed for the same HER current density in the cases of np‐Mo_2_C and commercial Mo_2_C, respectively. Given that MoS_2_ catalysts that are well known for high activity in the HER manifested the overpotentials of 166 and 253 mV in amorphous and crystalline phases, respectively, under the same experimental condition (Figure S4, Supporting Information), the activity of np‐Mo_2_C seemed to be within the comparable range to other conventional Mo compound catalysts. To further investigate the performances of commercial and the electrochemically synthesized Mo_2_C, the loaded amount of Mo carbide on np‐Mo_2_C was calculated by multiplying the geometric area of the electrode (0.196 cm^2^), thickness of the carbide film (2 µm), and density of Mo_2_C (8.9 g cm^−3^) under the assumption of zero‐porosity. Given that np‐Mo_2_C has a nanoporous morphology, no more than 349 µg of electrochemically synthesized Mo carbide is present on the electrode for HER measurement. Meanwhile, the weight of the commercial Mo_2_C loaded on the rotating disk electrode (RDE) was 491 µg over the same geometric area (0.196 cm^2^). Therefore, we were able to verify that the performance of np‐Mo_2_C is superior to that of commercial Mo_2_C, and this result is ascribable to the effective utilization of active materials by favorable nanostructures and excellent interconnections in np‐Mo_2_C, as discussed in the previous section.

It is also noteworthy that the np‐Mo_2_C is superior in performance even with the graphitic carbon shell with ≈1 nm thickness on the surface. We additionally performed ADT of the Mo carbides over 3000 potential cycles, and the HER activities of commercial Mo_2_C and np‐Mo_2_C after the ADT are displayed as dashed lines in Figure 7a. Unlike commercial Mo_2_C, which shows a poor stability for long‐term operation, np‐Mo_2_C exhibits a negligible performance drop, even after 3000 cycles of ADT. The highly durable properties of np‐Mo_2_C can be understood as a consequence of excellent connectivity, exclusion of polymeric binders and carbonaceous conducting agents, and presence of thin carbon shells on the surface. The HER activity of commercial Mo_2_C without a conducting agent was also measured for comparison, but extremely poor performance and stability was observed (Figure S5, Supporting Information).

The Tafel slopes of the HER electrocatalysts were obtained by linear fits within the kinetic region, and the values are displayed in Figure 7b. It is well known that the Tafel slopes of 30, 40, or 120 mV dec^−1^ indicate that the rate determining step is Tafel (H_ads_ + H_ads_ → H_2_), Heyrovský (H^+^ + e^−^ + H_ads_ → H_2_), or Volmer (H^+^ + e^−^ + * → H_ads_, * stands for an active site) reaction, respectively.^68^, ^69^, ^70^ The Tafel slope of the Pt foil is measured as 35.0 mV dec^−1^, which is consistent with previous reports that the reaction between H_ads_ dictates the overall HER performance for Pt.^68^, ^71^ On the other hand, both commercial and electrochemically synthesized Mo carbide exhibit Tafel slopes of ≈100 mV dec^−1^, which shows that the HER proceeds on Mo_2_C through Volmer–Heyrovský reaction. We additionally obtained the HER polarization curve and Tafel plot of np‐MoO_3_, and the results are displayed in Figure S6 in the Supporting Information. The activity of np‐MoO_3_ is inferior to those of the Mo carbides, and it manifests a Tafel slope of 107.9 mV dec^−1^ in the kinetic current region. This indicates that the HER mechanism on the surface of MoO_3_ in an acidic medium also follows Volmer–Heyrovský steps. Meanwhile, the Tafel slope of np‐MoO_3_ shows a steep increase at ≈−0.18 V versus RHE, indicating that hydrogen adsorption is more severely hindered at lower potentials. Mo oxide is a well‐known material for rapid cation intercalation,^72^, ^73^, ^74^ and thus the rate limiting at hydrogen adsorption reaction can be attributed to the competition between proton intercalation and hydrogen adsorption. There are possibilities for H^+^ located near the Mo oxide to move into the oxide lattice rather than participating in Volmer reaction, consequently deteriorating the adsorption of hydrogen.

### 2.4 Investigations on the Role of Carbon Shells

**Figure 8:**
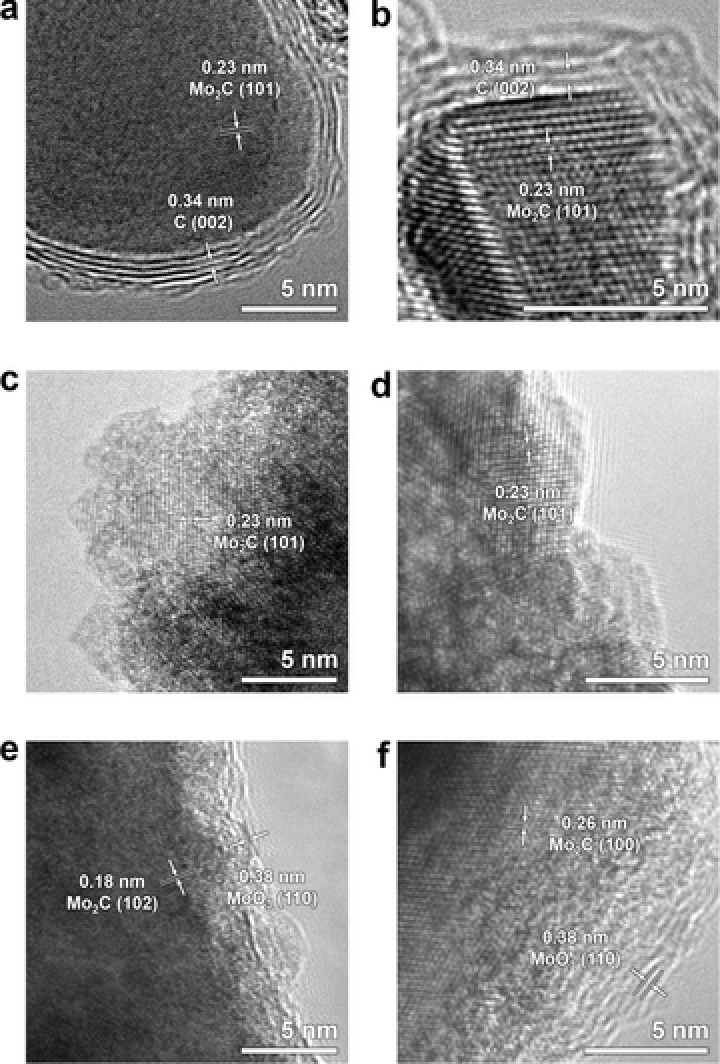
a,b) TEM images of electrochemically synthesized Mo carbide after the ADT, and those of commercial Mo_2_C c,d) before and e,f) after the ADT.

To understand the origin of the stability of np‐Mo_2_C, TEM analyses were performed on the electrochemically synthesized and commercial Mo_2_C after the ADT. **Figure** **8**a,b shows high‐magnification TEM images of np‐Mo_2_C after 3000 cycles of accelerated potential scans, and no notable changes are observed at the surface of Mo_2_C. In contrast, severe surface oxidation is apparent in commercial Mo_2_C after the durability test. TEM images were also obtained for the commercial Mo_2_C, and they are displayed in Figure 8c–f (high magnification) and Figure S7 in the Supporting Information (low magnification). Figure 8c,d shows the TEM images of the commercial Mo_2_C before the potential cycling; the Mo_2_C lattice is clearly observable even at the outermost surface. However, Mo oxide layers form on the surface of the commercial Mo_2_C (Figure 8e,f) after 3000 cycles of ADT. The Mo oxide is identified at the surface of the commercial Mo_2_C based on the d‐spacing of 0.38 nm, which is consistent with the (110) plane of MoO_3_.

**Figure 9:**
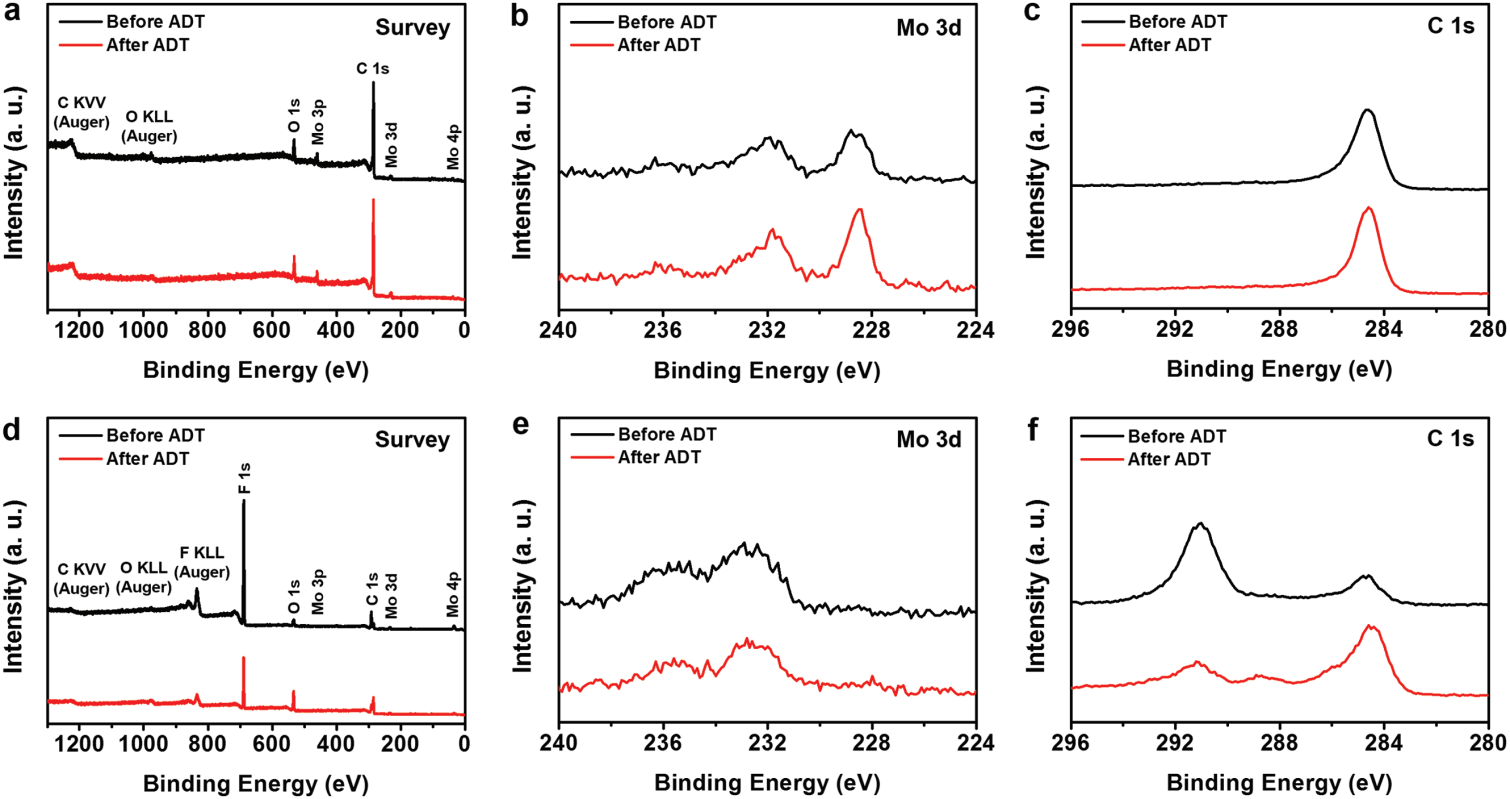
XPS a,d) survey, b,e) Mo 3d, and c,f) C 1s spectra of a–c) electrochemically synthesized Mo carbide and d–f) commercial Mo_2_C before and after the ADT.

The changes in surface states of the Mo carbide catalysts during the electrochemical H_2_ production were additionally investigated by ex situ XPS analyses before and after the ADT. **Figure** **9**a,d shows survey spectra of np‐Mo_2_C and commercial Mo_2_C, respectively, and only Mo, C, and O signals are observed, except for a strong F 1s peak for commercial Mo_2_C. The signal related to F originates from the polymeric Nafion binder used for the catalyst ink preparation and drop casting. The core level Mo 3d (Figure 9b) and C 1s (Figure 9c) spectra of np‐Mo_2_C indicate a negligible change in the chemical states of the surface during the 3000 cycles of ADT. Meanwhile, as can be seen from the Mo 3d spectra displayed in Figure 9e, strong Mo oxide signals located at 232.7 and 235.9 eV were observable for commercial Mo_2_C both before and after the potential cycling. This is ascribable to surface oxidation of the commercial carbides that occur upon contact with air or during the catalyst ink preparation, and we could not obtain any useful information from the Mo 3d spectra of the commercial Mo_2_C catalysts. On the other hand, a significant difference is observed between the C 1s spectra (Figure 9f) of the commercial Mo_2_C obtained before and after the ADT. Signals at 284.6 eV show the presence of graphitic carbon, and the peaks at ≈291 eV originate from the C–F species in the Nafion binders. The decreased intensity of signals from C–F species (in C 1s and F 1s) and the increase in the peak from graphitic carbon might be the consequence of degradation or detachment of binders. However, given that the Nafion is a widely used binder in aqueous electrocatalysis due to their excellent stability,^75^ the difference between the intensities of the peaks at around 291 and 284.6 eV seems to be ascribable to the inhomogeneity in the catalyst ink, though it was prepared with the assistance of ultrasonication. Interestingly, a peak at around 289 eV is apparent for the commercial Mo_2_C after the ADT, while it was undetectable beforehand. According to the results from previous studies, this peak is understood as the signals from carbonyl species that form on Mo_2_C during surface oxidation.^76^, ^77^ These results verify that chemical oxidation of Mo_2_C occurs during the electrochemical HER, although this phenomenon is not observed for np‐Mo_2_C. This implies that the carbon shells on the surface of the catalysts play a key role in enhancing the durability by suppressing surface oxidation of Mo carbide catalysts.

**Figure 10:**
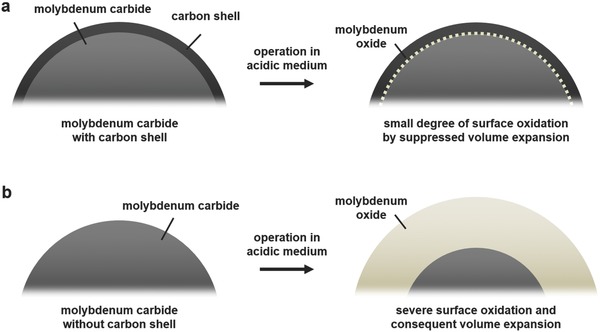
Schematic images of the surface oxidation of a) Mo carbide with a carbon shell on the surface and b) bare Mo carbide before and after the electrochemical operation in an acidic medium.

The discussion above leaves an unanswered question on the role of carbon shells on the stability of np‐Mo_2_C because the surface of Mo carbide beneath the carbon shell does participate in the electrochemical HER; this contrasts the benefits of carbon shells that are usually understood as a layer that blocks the surface from chemical degradation. However, there is an inconsistency here because the catalytic reaction and chemical oxidation occur on the same surface of the electrocatalysts. For that reason, the exact role of carbon shells on the surface of various electrocatalysts is still unclear, though their effectiveness in terms of enhanced durability has been experimentally verified in a number of applications.^44^, ^45^, ^64^, ^65^, ^66^, ^67^ Based on our observations from the ex situ analyses, we would like to suggest here that the role of carbon shells is to serve as mechanical barriers to suppress oxidation of carbide surface that unavoidably accompanies volume expansion, as described in the schematic images in **Figure** **10**. Unlike Mo_2_C, wherein there are two Mo atoms per carbon atom, three O atoms exist per Mo atom in MoO_3_. This difference creates a significant gap between the volume of Mo_2_C and that of MoO_3_, which indicates that oxidation of Mo carbide accompanies a sharp increase in volume. Therefore, thin carbon shells may suppress surface oxidation by their mechanical strength that hinders volume expansion of the carbide catalysts. This enables a general explanation on the role of carbon shells without any contradiction because our suggestion explains the suppression of chemical oxidation without blocking of active sites of electrocatalysts. We believe that our suggestion provides a possible origin for the excellent durability of np‐Mo_2_C and gives important insights into comprehending the origin of the enhanced durability of electrocatalysts with carbon shells because surface oxidation is one of the most dominant degradation mechanisms in various compound and metallic electrocatalysts.^78^, ^79^

## 3 Conclusions

In summary, we prepared nanostructured Mo carbide by electrochemical anodization of Mo foil followed by heat treatment in a CO atmosphere. During the anodization, a distinctive behavior of Mo was observed, whereby exfoliation of metallic Mo at the (110) planes occurred rather than formation of nanostructured anodic oxides. The exfoliated Mo nanosheets were viable for postmodification, and Mo carbide with a nanoporous morphology and excellent interconnections was prepared. When applied to the HER, the Mo carbide electrode manifested a highly active and durable performance as an electrocatalyst and exhibited a negligible performance drop after 3000 cycles of accelerated operations. Furthermore, the role of carbon shells on the surface of the Mo carbide was suggested as a mechanical barrier to suppress oxidative degradation at the catalyst surface, which inevitably accompanies volume expansion. We believe that the findings and discussion performed in this study provide important insights into the design and synthesis of nanostructured Mo compound catalysts for the HER. Moreover, our understanding on the role of the thin carbon shells is anticipated to bring advances in the durability of nanoelectrocatalysts for practical and long‐term operation of electrochemical devices.

## 4 Experimental Section

*Preparation and Physical Characterization of Nanoporous MoO_3_ and Mo_2_C*: Molybdenum foil (99.95%, 0.25 mm thick, Alfa Aesar) was cut into 1.5 cm × 2.0 cm pieces, flattened, and sequentially cleaned in acetone, ethanol, and deionized water for 10 min by ultrasonication. Electrochemical anodization of Mo foil was performed in a two‐electrode system at 40 V for 2 h at 25 °C using an ethylene glycol electrolyte containing 0.25 wt% NH_4_F, 0.1 m NaOH, and 2 vol% H_2_O, and a Pt mesh was used as the counter electrode. After anodization, crystalline MoO_3_ and Mo_2_C films were prepared by heat treatment at 450 °C for 4 h in air and at 800 °C for 4 h in a CO atmosphere, respectively. The commercial Mo_2_C electrode was prepared by loading Mo_2_C powders (99.5%, Alfa Aesar) on a glassy carbon by casting in the form of ink with the assistance of Nafion binder and carbon black as a conducting agent. For the comparative study, commercial crystalline MoS_2_ (99%, Sigma‐Aldrich) and amorphous MoS_2_ synthesized according to a previous report^80^ were used. The morphologies of the Mo compounds were imaged by using SEM (Carl Zeiss AURIGA) and TEM (JEOL JEM‐2100F). The XRD measurements were performed using a Rigaku D‐MAX2500‐PC, and the XPS spectra were obtained using Thermo SIGMA PROBE spectrometer. Raman spectra were measured using Horiba Jobin‐Yvon LabRam Aramis spectrometer (excitation source: 514 nm line of an Ar‐ion laser).

*Electrochemical Analyses*: Polarization curves for the HER were obtained using an RDE at a rotation speed of 2000 rpm. Nanoporous Mo_2_C was attached to a specially designed RDE tip, and commercial Mo_2_C powders mixed with Nafion (as a binder) and carbon black (as a conducting agent) were loaded onto the glassy carbon tip for RDE measurements. Potential cycling was performed using a potentiostat (Metrohm Autolab PGSTAT). A glassy carbon rod served as the counter electrode and a saturated calomel electrode was used as a reference during the measurements. H_2_‐saturated 0.5 m H_2_SO_4_ solution was used as the electrolyte, and the scan rates were 20 and 100 mV s^−1^ for obtaining HER polarization curves and ADT cycling, respectively. All the electrochemical measurements were performed at 293 K. For the ex situ analysis of the commercial Mo_2_C before and after the ADT, fluorine‐doped tin oxide‐coated glass (TEC‐8, Pilkington) was used as a conductive substrate.

## Conflict of Interest

The authors declare no conflict of interest.

## Supporting information

Supplementary
