# Supplementary material for: Electrochemically Synthesized Nanoporous Molybdenum Carbide as a Durable Electrocatalyst for Hydrogen Evolution Reaction
Source: Adv Sci (Weinh). 2017 Dec 19;5(1):1700601. doi: 10.1002/advs.201700601 (PMC5770677; doi:10.1002/advs.201700601)
Supplement: Supplementary file 1 — Supplementary [file ADVS-5-na-s001.pdf]

Copyright WILEY-VCH Verlag GmbH & Co. KGaA, 69469 Weinheim, Germany, 2017.

ADVANCED SCIENCE
Open Access

Supporting Information

for *Adv. Sci.*, DOI: 10.1002/advs.201700601

Electrochemically Synthesized Nanoporous Molybdenum Carbide as a Durable Electrocatalyst for Hydrogen Evolution Reaction

Jin Soo Kang, Jin Kim, Myeong Jae Lee, Yoon Jun Son, Dong Young Chung, Subin Park, Juwon Jeong, Ji Mun Yoo, Heejong Shin, Heeman Choe, Hyun S. Park, and Yung-Eun Sung**

Copyright WILEY-VCH Verlag GmbH & Co. KGaA, 69469 Weinheim, Germany, 2016.

Supporting Information

Electrochemically Synthesized Nanoporous Molybdenum Carbide as a Durable Electrocatalyst for Hydrogen Evolution Reaction

Jin Soo Kang, Jin Kim, Myeong Jae Lee, Yoon Jun Son, Dong Young Chung, Subin Park, Juwon Jeong, Ji Mun Yoo, Heejong Shin, Heeman Choe, Hyun S. Park, and Yung-Eun Sung**

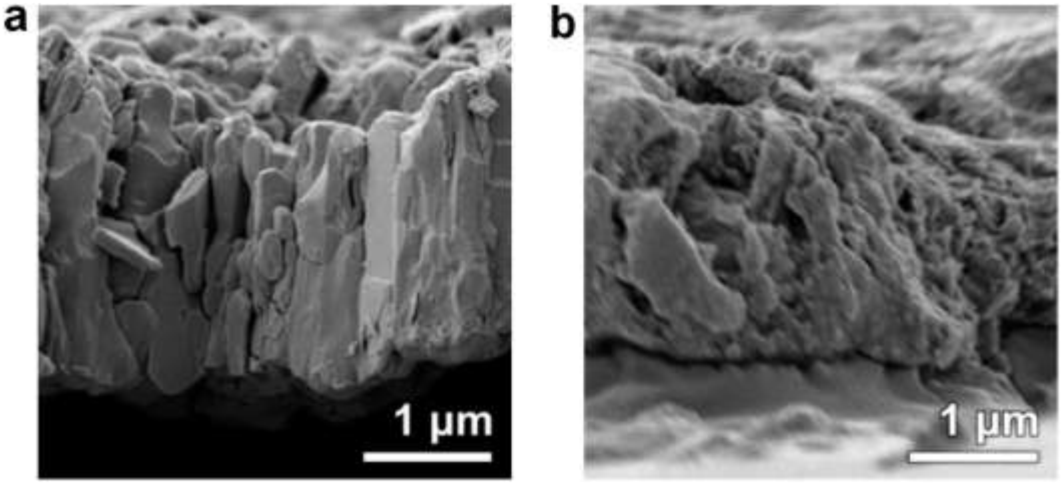

Figure S1. Cross-sectional SEM images of (a) Mo oxide and (b) Mo carbide prepared by electrochemical anodization followed by post heat treatments.

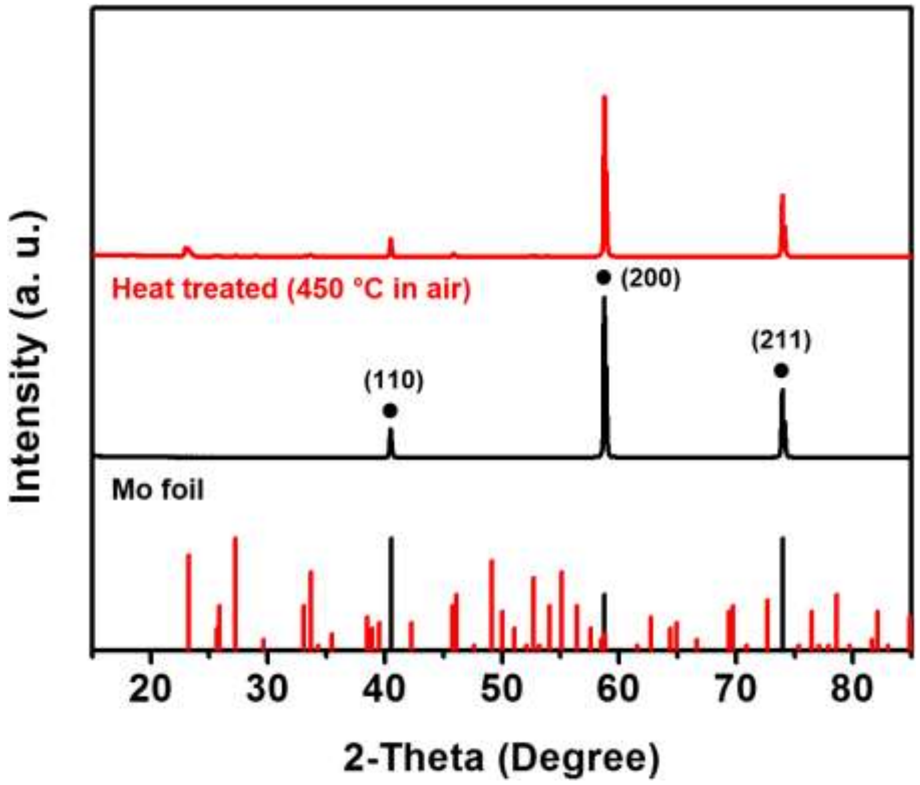

Figure S2. XRD patterns of Mo foil and heat-treated Mo foil at 450 °C in air for 4 h. The signals were assigned in accordance with the reference 2-theta positions of Mo (black bars, JCPDS 01-1208) and MoO_3 (red bars, JCPDS 05-0506).

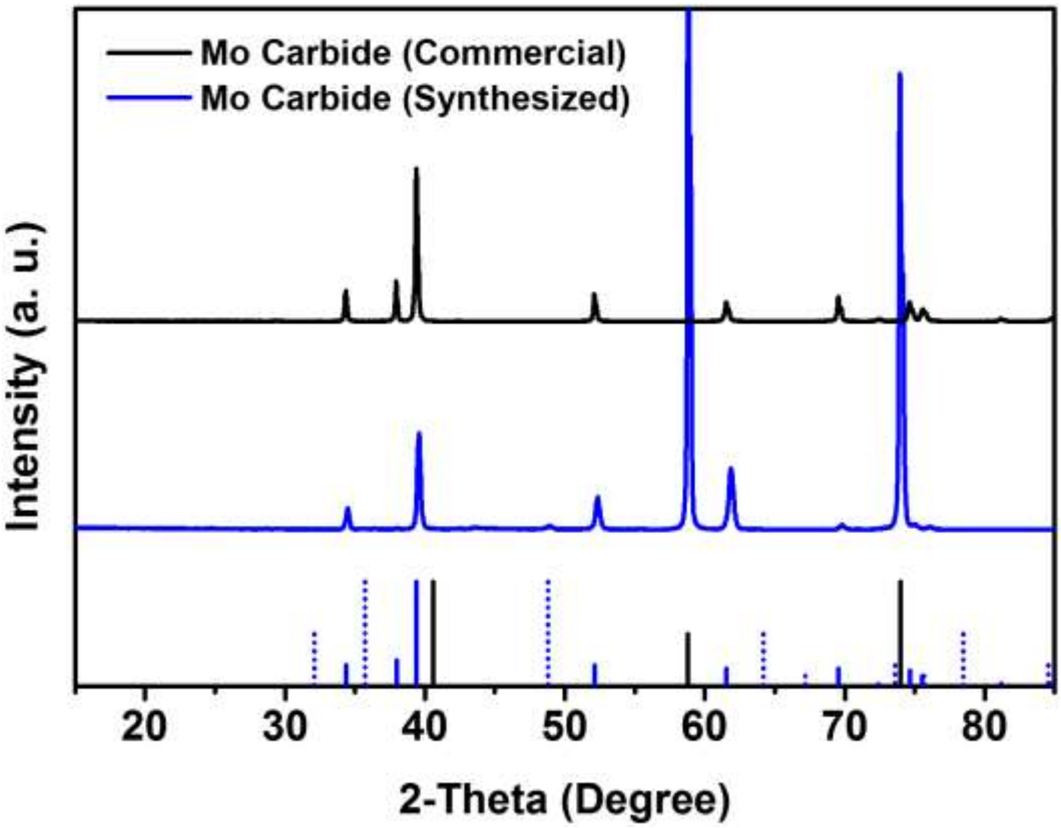

Figure S3. XRD patterns of np-Mo_2C and commercial Mo_2C. The signals were assigned in accordance with the reference 2-theta positions of Mo (black bars, JCPDS 01-1208), MoC (blue dotted bars, JCPDS 45-1015), and Mo_2C (blue bars, JCPDS 35-0787).

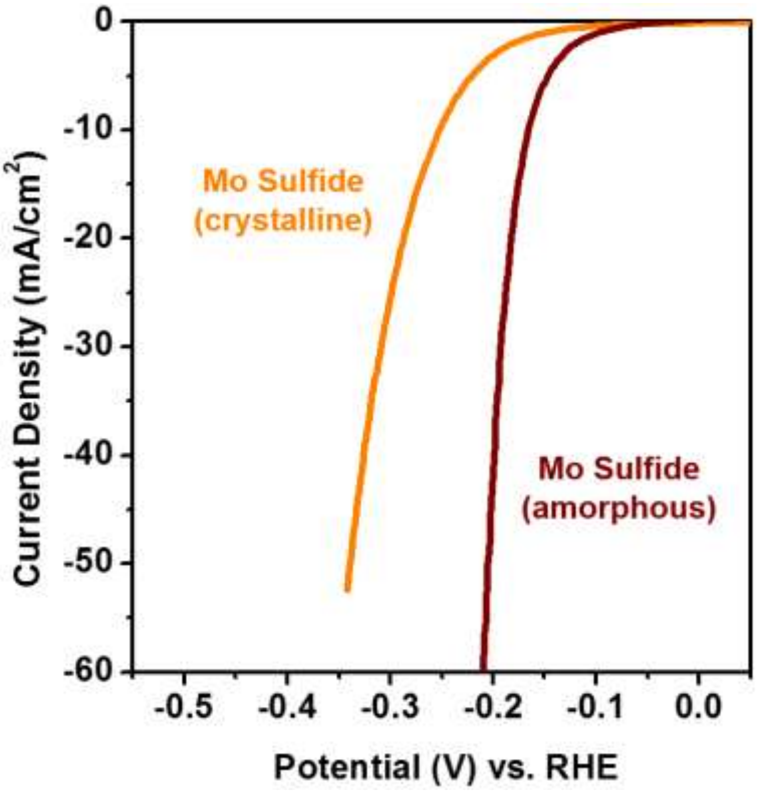

Figure S4. iR-corrected HER polarization curves of crystalline and amorphous MoS_2 measured with the presence of carbon black conducting agents. The amount of loaded catalyst was 424 μg for both, and the geometric area of the RDE was 0.196 cm^2.

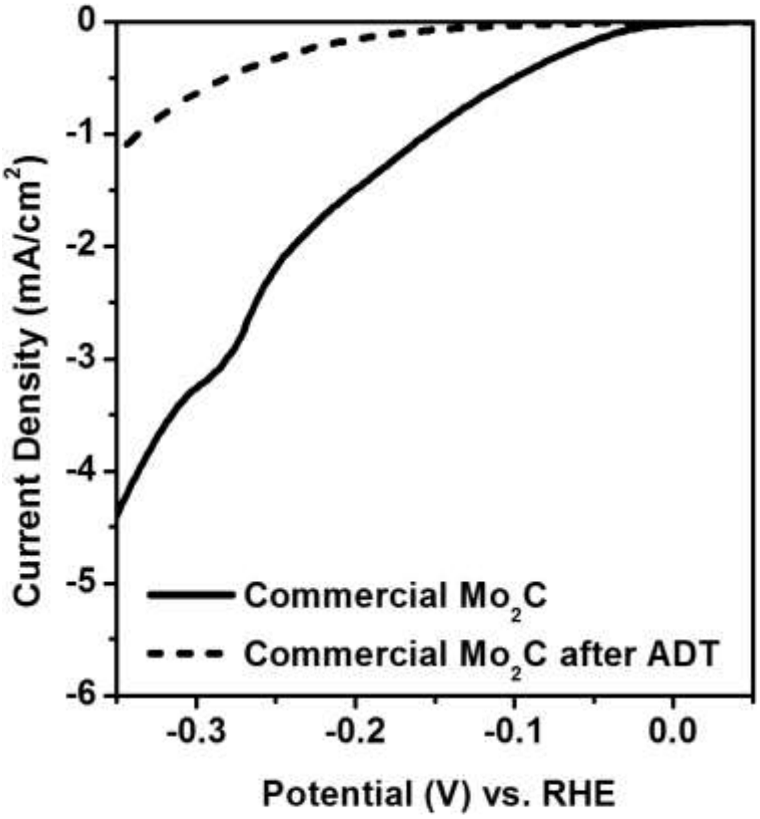

Figure S5. iR-corrected HER polarization curves of commercial Mo_2C measured without the assistance of conducting agents. The amount of loaded catalyst was 972 μg, and the geometric area of the RDE was 0.196 cm^2.

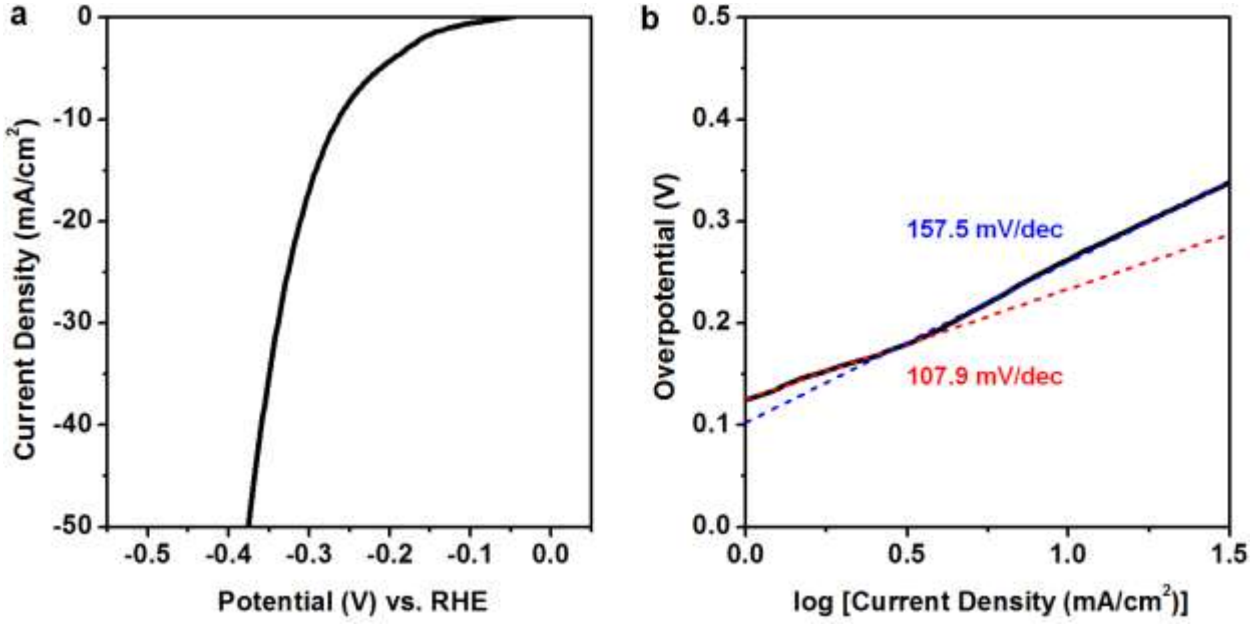

Figure S6. (a) iR-corrected HER polarization curve and (b) Tafel plot of np-MoO_3. The dashed lines in (b) are linear fits of the Tafel plot in different ranges of current density. The values noted on (b) show the Tafel slopes.

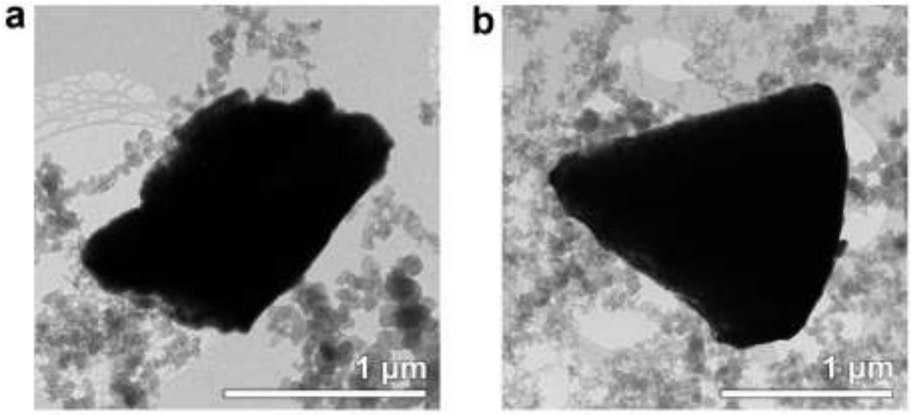

Figure S7. TEM images of commercial Mo_2C (a) before and (b) after the ADT. The small particles shown in the images are the Vulcan carbons added to the catalyst ink to improve the electrical conductivity during the electrochemical measurements.
